# Inhibitory Control Deficits Associated with Upregulation of CB_1_R in the HIV-1 Tat Transgenic Mouse Model of Hand

**DOI:** 10.1007/s11481-019-09867-w

**Published:** 2019-08-01

**Authors:** Ian R. Jacobs, Changqing Xu, Douglas J. Hermes, Alexis F. League, Callie Xu, Bhupendra Nath, Wei Jiang, Micah J. Niphakis, Benjamin F. Cravatt, Ken Mackie, Somnath Mukhopadhyay, Aron H. Lichtman, Bogna M. Ignatowska-Jankowska, Sylvia Fitting

**Affiliations:** 1 Department of Psychology & Neuroscience, University of North Carolina at Chapel Hill, Chapel Hill, NC 27599 USA; 2 Department of Chemistry & Biochemistry, North Carolina Central University, Durham, NC 27707 USA; 3 Department of Microbiology and Immunology, Medical University of South Carolina, Charleston, SC 29425 USA; 4 Division of Infectious Diseases, Department of Medicine, Medical University of South Carolina, Charleston, SC 29425 USA; 5 The Skaggs Institute for Chemical Biology, Department of Chemistry, The Scripps Research Institute, La Jolla, CA 92037 USA; 6 Department of Psychological & Brain Sciences, Indiana University, Bloomington, IN 47405 USA; 7 Department of Pharmacology & Toxicology, Virginia Commonwealth University, Richmond, VA 23298 USA; 8 Neuronal Rhythms in Movement Unit, Okinawa Institute of Science and Technology, Okinawa, 904-0495 Japan

**Keywords:** Transactivator of transcription, Go/no-go task, Cannabinoid receptor type-1, Endocannabinoids, FAAH enzyme inhibition, Infralimbic cortex

## Abstract

In the era of combined antiretroviral therapy, HIV-1 infected individuals are living longer lives; however, longevity is met with an increasing number of HIV-1 associated neurocognitive disorders (HAND) diagnoses. The transactivator of transcription (Tat) is known to mediate the neurotoxic effects in HAND by acting directly on neurons and also indirectly via its actions on glia. The Go/No-Go (GNG) task was used to examine HAND in the Tat transgenic mouse model. The GNG task involves subjects discriminating between two stimuli sets in order to determine whether or not to inhibit a previously trained response. Data reveal inhibitory control deficits in female Tat(+) mice (*p* = .048) and an upregulation of cannabinoid type 1 receptors (CB_1_R) in the infralimbic (IL) cortex in the same female Tat(+) group (*p* < .05). A significant negative correlation was noted between inhibitory control and IL CB_1_R expression (*r* = −.543, *p* = .045), with CB_1_R expression predicting 30% of the variance of inhibitory control (*R*^*2*^ = .295, *p* = .045). Furthermore, there was a significant increase in spontaneous excitatory postsynaptic current (sEPSC) frequencies in Tat(+) compared to Tat(−) mice (*p* = .008, across sexes). The increase in sEPSC frequency was significantly attenuated by bath application of PF3845, a fatty acid amide hydrolase (FAAH) enzyme inhibitor (*p* < .001). Overall, the GNG task is a viable measure to assess inhibitory control deficits in Tat transgenic mice and results suggest a potential therapeutic treatment for the observed deficits with drugs which modulate endocannabinoid enzyme activity.

**Electronic supplementary material:**

The online version of this article (10.1007/s11481-019-09867-w) contains supplementary material, which is available to authorized users.

**Graphical Abstract:**
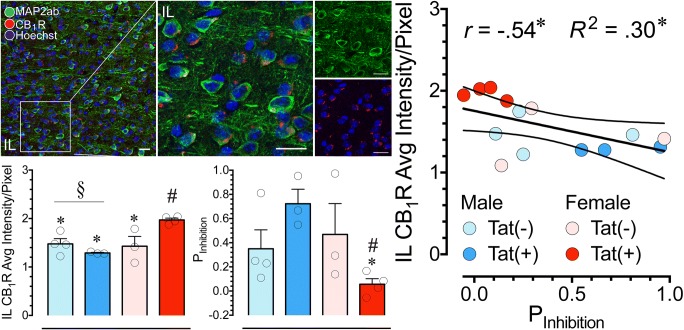
Results of the Go/No-Go operant conditioning task reveal inhibitory control deficits in female transgenic Tat(+) mice without significantly affecting males. The demonstrated inhibitory control deficits appear to be associated with an upregulation of cannabinoid type 1 receptors (CB_1_R) in the infralimbic (IL) cortex in the same female Tat(+) group.

## Introduction

The World Health Organization estimates 36.7 million people are diagnosed with human immunodeficiency virus type 1 (HIV-1) worldwide, which has been considered a fatal diagnosis before antiretroviral treatments were established. With the advent of combined antiretroviral therapy (cART), HIV-1 infection has become a chronic but manageable condition with decreased mortality rates and increased life expectancies (Harrison et al. 2010). Before cART, the later stages in the progression of HIV-1 infection were marked with HIV-1 associated dementia (HAD), but a newer, milder form of cognitive impairment has emerged during the post-cART era called HIV-1-associated neurocognitive disorders (HAND) (Ellis et al. 2007). Symptoms that have been associated with HAND in the post-cART era include reduced mental acuity, and deficits in working memory, attention, and inhibitory control (Connolly et al. 2014; Ernst et al. 2003; Wang et al. 2017) that are specifically associated with cortical brain structures such as the prefrontal cortex (PFC) (Heaton et al. 2011).

It is unclear how HAND manifests and what molecular and cellular mechanisms are driving HAND, however the severity of synaptic disruption and dendritic injury is correlated with the severity of expressed symptoms of HAND (Everall et al. 1999; Masliah et al. 1997). As the virus itself does not directly infect neurons, neuronal damage and synaptodendritic injury occurs indirectly through the release of toxic substances from infected microglia and other non-neuronal cells in the central nervous system (CNS), including viral proteins, cytokines/chemokines, and free radicals. Among the viral proteins, the transactivator of transcription (Tat) in particular plays a key role in HAND pathogenesis as Tat continues to be detected in the HIV-1 infected brain despite cART (Johnson et al. 2013; Mediouni et al. 2012). Further, expression of the Tat protein in transgenic animal models, without the presence of the virus itself or other viral proteins, tend to mirror the neuropathology and cognitive deficits observed in HIV-1 patients with HAND (Bruce-Keller et al. 2008; Carey et al. 2012; Fitting et al. 2010; Hauser et al. 2009; Kesby et al. 2014; Kesby et al. 2015; Marks et al. 2016; Maung et al. 2014; Mediouni et al. 2015; Paris et al. 2014a; Paris et al. 2014c; Toggas et al. 1994). Tat exerts its effects via multiple mechanisms, including direct and indirect effects on neurons (Chen et al. 1997; Cheng et al. 1998; El-Hage et al. 2008; El-Hage et al. 2005; Kutsch et al. 2000; Lipton 1993; Liu et al. 2000; Magnuson et al. 1995; Nath 2002). Tat can depolarize neurons directly by interacting with a variety of surface receptors, such as NMDA receptors (Eugenin et al. 2007; Longordo et al. 2006; Raybuck et al. 2017), and potentiate glutamate-induced excitotoxicity (Longordo et al. 2006), leading to increases in [Ca^2+^]_i_, and dendritic structural and functional defects in neurons (Fitting et al. 2014; Green et al. 2018; Haughey et al. 1999; Mattson et al. 2005). Additionally, Tat can induce neurotoxicity through indirect mechanisms via its actions on microglia and astrocytes by stimulating the production of proinflammatory cytokines (Chen et al. 1997), inducing TNF-α and IL-1 in monocytes and macrophages and a milieu of cytokines and chemokines in astrocytes, including IL-8, RANTES, MCP-1, and TNF-α (El-Hage et al. 2005; Kutsch et al. 2000).

One avenue for treating the effects of HIV-1 Tat in the brain is the modulation of the endocannabinoid (eCB) system. Modulating function of endogenous cannabinoids emerges as a promising therapeutic target in several neurodegenerative diseases due to their anti-excitotoxic and anti-inflammatory properties (Pertwee 2014; Scotter et al. 2010). Endocannabinoids have been reported to be up-regulated in disorders, such as Parkinson’s disease and Alzheimer’s disease, and reduce or abolish unwanted effects of these disorders or slow their progression (Pertwee 2014; Scotter et al. 2010). Interestingly, an upregulation of expression of cannabinoid type 1 and type 2 receptor levels (CB_1_R and CB_2_R, respectively) has been demonstrated in the CNS of HIV-1-infected individuals (Cosenza-Nashat et al. 2011) and in simian immunodeficiency virus (SIV) encephalitis (Benito et al. 2005). There is support for existing cannabinoid signaling pathways that can decrease neuronal injury, including CB_1_R activation as well as inhibition of NMDAR-mediated calcium influx (Liu et al. 2009). Multiple studies have indicated that CB_1_R stimulation limits synaptic excitation mediated by glutamate with CB_1_R activation decreasing glutamate-mediated excitatory postsynaptic currents (EPSCs) (Andre et al. 2010; Chevaleyre et al. 2006; Harkany et al. 2008; Marsicano et al. 2003; Monory et al. 2006; Rossi et al. 2011). Nevertheless, therapeutic use of direct CB_1_R agonists is limited due to the pervasive psychoactive side effects associated with CB_1_R agonists that include sensorimotor, affective and cognitive disturbances (Di Marzo 2008). Thus, research efforts have focused on development of drugs targeting components of the endogenous cannabinoid system, including enzymes regulating the biosynthesis and degradation of the two major endogenous cannabinoids N-arachidonoylethanolamine (anandamide/AEA) and 2-arachidonoylglycerol (2-AG) (Ahn et al. 2008; Lichtman et al. 2010; Petrosino and Di Marzo 2010). There is strong preclinical evidence that selective inhibitors of the main AEA-metabolizing enzyme, fatty acid amide hydrolase (FAAH), can ameliorate the unwanted effects in a variety of different laboratory animal models of neurodegenerative diseases (Naidoo et al. 2011; Pertwee 2014). The new generation of hydrolytic enzyme inhibitors, such as the FAAH enzyme inhibitor PF3845, has demonstrated highly improved selectivity, potency and produce less side effects compared to previously available compounds (Ahn et al. 2009; Booker et al. 2012; Ignatowska-Jankowska et al. 2015; Ignatowska-Jankowska et al. 2014; Niphakis et al. 2013; Parker et al. 2015). In a recent in vitro study we have demonstrated that PF3845 is protective against HIV-1 Tat-induced excitotoxicity and neuronal injury by involving CB_1_R and CB_2_R-mediated mechanisms (Hermes et al. 2018; Xu et al. 2017).

The aim of the present study was (1) to determine the effects of Tat on inhibitory control by using the PFC-related operant conditioning Go/No-Go (GNG) task, (2) to assess the effects of Tat on the eCB system by quantifying the changes in CB_1_R expression in the medial PFC (mPFC) using immunohistochemistry, and (3) to investigate the potential neuroprotective effects of the FAAH enzyme inhibitor PF3845 on Tat-induced increases in glutamatergic neurotransmission ex vivo. Results revealed inhibitory control deficits in female Tat(+) mice that also demonstrated an upregulation of CB_1_R expression in the infralimbic cortex (IL). A significant negative correlation between inhibitory control and IL CB_1_R expression demonstrated that deficits in inhibitory control were associated with an upregulation of IL CB_1_R expression, with IL CB_1_R expression predicting 30% of the variance of inhibitory control. Further, Tat-induced increases in spontaneous excitatory postsynaptic current (sEPSC) frequencies (females and males) were attenuated by application of PF3845, indicating a potential role of the eCB system in the context of HAND.

## Methods

### Subjects

Doxycycline (DOX)-inducible, brain-specific HIV-1_IIIB_ Tat_1–86_ transgenic mice were developed on a C57BL/6J hybrid background as described in detail in previous literature (Bruce-Keller et al. 2008; Hahn et al. 2015b). Tat expression, which is under the control of a tetracycline-responsive promoter controlled by glial fibrillary acidic protein (GFAP) expression, was induced with a specially formulated chow containing 6 mg/g DOX (product TD.09282; Harlan, Indianapolis, IN). Inducible Tat(+) transgenic mice express both *GFAP-rtTA* and *TRE-tat* genes, while control Tat(−) transgenic mice express only the *GFAP-rtTA* genes. At ~4 weeks of age transgenic mice were genotyped to confirm the presence of Tat and/or *rtTA* transgenes.

In all experiments animals were counterbalanced for sex within groups. For the behavioral and immunohistochemistry studies, adult transgenic mice (8 weeks of age) were experimental naive and included 7 Tat(+) mice (4 female) and 7 Tat(−) mice (3 female). To induce the Tat protein animals were fed DOX at ~6 weeks of age and kept on this diet until sacrificed for immunohistochemistry experiments (more than 10 months of DOX treatment). For the western blot and electrophysiology studies a new set of adult Tat transgenic mice [Tat(−) and Tat(+), DOX exposure for more than 5 months and at least ~1 month, respectively] was used with at least 6–8 mice in each group (3–4 females/per group). All animals were bred by the University of North Carolina Division of Comparative Medicine (UNC DCM) and individually housed (starting at 6 weeks of age) under a 12/12 h light-dark (LD) cycle. The colony room temperature was maintained at 21 °C and 32% humidity. All animal procedures were approved by the University of North Carolina Institutional Animal Care and Use Committee (IACUC) and are in keeping with AAALAC guidelines.

### Apparatus

Standard mouse experimental chambers (MED Associates ENV-307 W) were housed in sound and light attenuating cubicles (MED Associates ENV-022MD). Each chamber was equipped with a curved five nose-poke wall (MED Associates ENV-115C) where subjects performed their responses. Each nose-poke contained an imbedded yellow LED to illuminate the port. A 28 V DC, 100 mA house light (MED Associates ENV-215 W) was mounted on the wall opposite of the curved five nose-poke wall and illuminated the chamber during each session. Sucrose pellets (BioServ Product# F06233 - Dustless Precision Pellets®) weighing 20 mg served as reinforcers and were delivered via a pellet dispenser (MED Associates ENV-203-20) into a receptacle (MED Associates ENV-303 W), which was illuminated by a receptacle light (MED Associates ENV-303RL). The pellet receptacle was located under the house light, opposite of the curved nose-poke wall. Behavioral testing occurred in a dark room illuminated by red fluorescent lighting. The testing room was kept at 22 °C room temperature with 30% humidity.

### Behavioral Training

Training took place over four phases adapted from procedures described in previous literature (Gubner et al. 2010; Loos et al. 2010): shaping the nose-poke response, shaping the go response, titrating the limited hold (LH), and testing. Unless otherwise stated, advancement from a phase was contingent on criteria requiring the subject to earn 40 reinforcers and maintain 80% accuracy on 2 consecutive days. Subjects advanced through the phases individually as they independently reached criteria. This advancement style ensured that all subjects received the same relative training and prevented overtraining. Additionally, the intertrial interval (ITI) used in all phases was 10 s. The house light was illuminated 1 s before the start of the trial and was terminated once the animal retrieved a reinforcer. All sessions terminated after 30 min or after 100 reinforcers were earned.

After individual housing placement, mice were allowed to acclimate to their new living conditions for one week and on the final two days, weights were taken and averaged to compute the initial weights from which the 85% target weight could be derived. At ~7 weeks of age, mice were gradually transitioned from ad libitum feeding to a restricted diet to lower their body weights to 85% of initial. At ~8 weeks of age mice entered the study and underwent 3 training phases (please see supplemental material for full description of the 3 training phases). Briefly, phase 1 started with magazine training in which a pellet was dispensed on a variable-time 2 min schedule to train the animal where reinforcement was delivered and familiarized them with the sound of a pellet being dispensed. Once subjects met criteria for this phase, they advanced to phase 2 where subjects were introduced to the chain of behaviors necessary to receive reinforcement on a Go trial on 80% of trials and the No-Go trial on 20% of trials. On a Go trial first the subject was required to make a single poke into a specified port to receive reinforcement. On a No-Go trial, the house light flashing served as the No-Go stimulus and required the subject to not poke into any ports to receive reinforcement. Once subjects met criteria for this phase, they advanced to phase 3, which proceeded exactly as phase 2 but collected the go reaction time (GoRT), which was the latency from when the Go stimulus illuminated to when the subject performed a nosepoke into that port. Here an individually titrated LH was introduced on the Go and No-Go stimuli, which required the subject to respond to the Go stimulus, or withhold a response in the presence of the No-Go stimulus, in the LH period equal to the 90^th^ percentile of their GoRT in order to receive reinforcement. By adding the individually titrated LH, we were able to record omissions to the Go stimulus, which was a necessary measure for calculating an index of inhibition. Subjects that met criteria for this phase advanced to the testing phase 4.

**Fig. 1:**
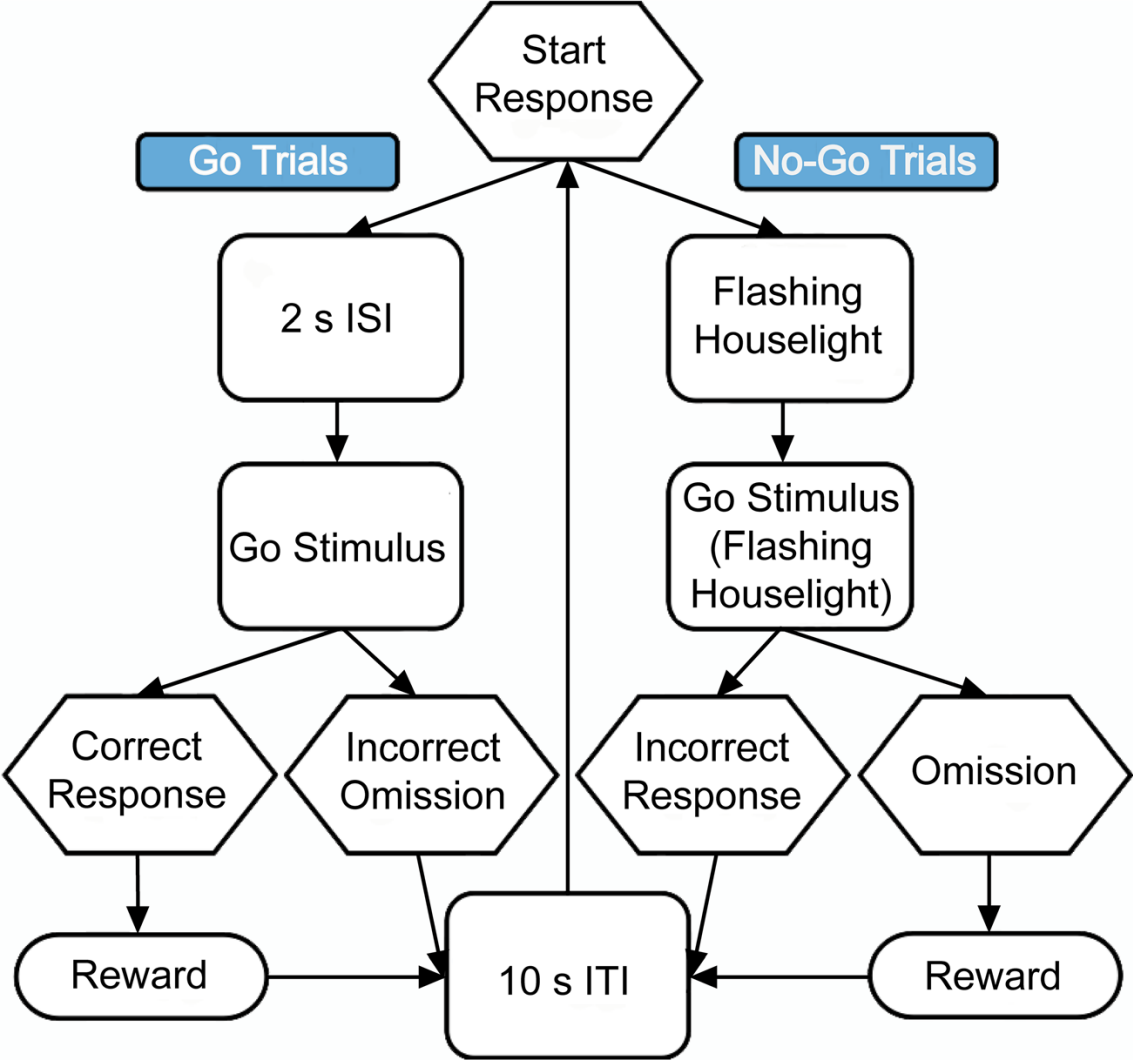
Demonstrates one of two trial arrangements of the Go/No-Go task at the testing phase that were used in this experiment. A trial begins with the Start stimulus being illuminated and the subject is required to make a response into the Start stimulus to begin the trial. On Go trials, the subject is required to wait for a 2 s inter-stimulus interval (ISI) before the Go stimulus will be presented. For both Go and No-Go arrangements, responses made into the Start stimulus during this time are counted as preservative responses and responses into the Go stimulus are counted as premature responses. Once the Go stimulus illuminates, the subject has until its individually titrated limited hold period lapses to make a response before that trial is scored as an incorrect omission. If the subject responded correctly, it receives a reward and waits for a 10 s intertrial interval (ITI) before a new trial begins. An incorrect omission yields no reward and the subject must wait for the 10 s ITI before a new trial commences. The No-Go trials proceeded very similarly, except during the 2 s ISI seen in Go trials a flashing house light is presented. After 2 s, the Go stimulus is illuminated and the house light continues to flash. The subject has until its individually titrated limited hold period lapses to make an incorrect response or the trial is counted as a correct omission. If the subject correctly omitted a response, it receives a reward before waiting for the 10 s ITI to start a new trial; otherwise, the subject simply waits for the 10 s ITI with no reward. Taken together, this setup presents a conditional discrimination in which the subject is required to alter responding depending on the status of the house light. For the other trial arrangements, the role of the house light is simply reversed where now a flashing house light indicates the subject should perform a response for reinforcement. These two arrangements are counterbalanced between groups and sexes to account for any fear inducing qualities of the flashing house light. There were no statistically significant differences between the two arrangements on any measure (all *p*’s > .05)

Phase 4 was the testing phase and occurred 10 months after DOX treatment. Trials presented during phase 4 are summarized in Fig. 1. The setup for this phase was the same as phase 3 with the exception that the No-Go trials were increased to 50% of the trials. During this phase, P_Inhibition_ was calculated from the number of omitted Go and No-Go trials. The formula is summarized below. The product of this formula is bound between 1 and − 1 where numbers closer to −1 indicate more inhibition on Go trials, numbers closer to 1 indicate more inhibition on No-Go trials, and numbers around 0 indicate a lack of discrimination between Go and No-Go trials. Test phase takes place on a single day and subjects were removed from the study once they complete their test phase.

$$ {P}_{Inhibition}=\frac{P_{No- go}-{P}_{Omission}}{1-{P}_{Omission}} $$

Note that P_No-Go_ indicates the proportion of correct omissions during No-Go trials to the total number of No-Go trials, whereas P_Omission_ indicates the proportion of incorrect omissions during Go trials to the total number of Go trials. Values closer to 0 indicate lower omissions than values closer to 1.

In addition to the P_Inhibition_, P_No-Go_, and P_Omission_ scores, perseverative and premature responses were tracked as indices of hyperactivity. A perseverative response was counted as any responses made into the Start stimulus after the initial response requirement is met. The total number of perseverative responses made on either trial type was combined and compared between subjects. A premature response was counted when the subject made a response into the Start or Go stimulus before that stimulus was illuminated. The total number of premature responses on either stimulus were combined and compared between subjects. Traditionally, premature responding has been a measure of impulsivity; however, studies interpreting this measure as impulsivity used timeouts in conjunction with a premature response. The task used here did not include timeouts, thus premature responses lacked a consequence and could not be interpreted as impulsivity. Finally, reinforcers earned and accuracy measurements on Go trials served as an index for motivation to earn reinforcers and ability to perform the basic trained operant response reliably.

### Immunohistochemistry

At the conclusion of behavioral testing, Tat transgenic mice were anesthetized with isoflurane and perfused using a 4% paraformaldehyde solution. Brains were extracted and post-fixed in 4% paraformaldehyde for 6 h at 4 °C before being washed 3x for 1 h at room temperature in 1x phosphate buffer solution (PBS) and incubated for 24 h at 4 °C in a 20% sucrose solution. Brains were then encapsulated in Tissue-Tek O.C.T compound before being frozen and kept at −80 °C until sectioning. Coronal sections were cut using a Leica CM3000 cryostat (Leica, Deerfield, IL) at −21 ± 1 °C to a width of 30 μm thickness. Sections were washed, and then treated with a permeability solution [0.1% Triton 100x, 0.1% bovine serum albumin (BSA) in PBS] for 15 min. Following, tissue sections were washed again before being exposed to a blocking solution (1% normal goat serum, 0.2% BSA in PBS) for 1 h. Sections were then incubated overnight at 4 °C in primary antibodies diluted in blocking solution against MAP2ab (mouse, Millipore, MAB378; 1:1000) and the rat CB_1_R-NH (raised to amino acids 1–77 of the N-terminus; rabbit; 1:500, Tsou et al. 1998). Next day, sections were washed again before being exposed to secondary antibodies conjugated to goat-anti-mouse Alexa 488 (Molecular Probes, O-6380, 1:500, green) for MAP2ab and goat-anti-rabbit Alexa 594 (Molecular Probes, A11012; 1:1000, red) for CB_1_R-NH. Secondary antibodies were diluted in the blocking solution previously described and applied for 1 h at room temperature. Following secondary application, tissue sections were washed in PBS and exposed to Hoechst (Hoechst 33342; Molecular Probes, H3570) for 3 min. Tissue sections were then washed and mounted on SuperFrost Plus Slides (Fisher Scientific, Pittsburgh, PA) using ProLong Gold antifade mounting media (Molecular Probes, P36930).

For imaging, eight tissue sections were chosen per animal that were representative of progression through the mPFC as per the Allen Mouse Brain Atlas (2004). Confocal immunofluorescent images for CB_1_R expression were gathered by an experimenter blinded to genotype and sex using a Zeiss LSM800 T-PMT laser scanning confocal microscope (Zeiss, Thornwood, NY) fitted with a 63x oil immersion objective. Images were acquired by using identical parameters for all groups (i.e., identical objective, zoom, laser intensity, gain, offset, and scan speed) optimized for control tissues. ZEN 2010 blue edition software (Carl Zeiss, Inc., Thornwood, NY) was used to collect the images. Four images were taken per tissue section (2 images from the left and 2 images from the right hemisphere), with unique fields of view for each section, one from the prelimbic (PL) and one from the infralimbic (IL) region of each hemisphere. The entire image was used as region of interest and processed using ImageJ (Schneider et al. 2012) to quantify the density of CB_1_R-NH staining per pixel in each image. Mean fluorescent intensities were determined with ImageJ without digital manipulation. For each animal, in each brain region (PL and IL), data are averaged across sections.

### Western Blot Analysis

Western blot analysis was carried out as previously described (Xu et al. 2017). In brief, tissue from the mPFC of Tat transgenic mice (DOX exposure for more than 5 months) was freshly harvested and homogenized on ice in an appropriate volume of ice-cold RIPA Lysis and Extraction buffer (G-Bioscience) with protease inhibitor cocktail (Amresco, Ohio). Blots were incubated with anti-CB_1_R (rabbit polyclonal; Cayman; 1:1000 dilution for 3 h), and anti β-Actin (anti-mouse from Cell Signaling; 1:1000 for 3 h) at room temperature. ECL reaction (Amersham) was used to dectect immunoreactive bands from the blot using BioRad Gel Doc XR+ system and image acquiring software (Image Lab ver. 5.1). Densitometric analysis used a modified version (version 1.59) of the Scion Image software (Scion Corporation).

### Electrophysiology

For the electrophysiology experiments brain slices of Tat transgenic mice (DOX exposure for at least ~1 month) were prepared following an established procedure as previously described (Xu et al. 2016). Mice were anesthetized with isofluorane and brains were removed after decapitation and submerged into ice-cold sucrose cutting solution containing in mM: 254 sucrose, 10 D-glucose, 26 NaHCO_3_, 2 CaCl_2_, 2 MgSO_4_, 3 KCl, and 1.25 NaH_2_PO_4_, saturated with 95% O_2_/5% CO_2_, at pH 7.4, 300 mOsm. Coronal brain slices (300 μm thickness) containing the mPFC were obtained using the VT 1000S microtome (Leica, Deerfield, IL). Slices were then incubated at 32 ± 1 °C for 30 min in artificial cerebrospinal fluid (aCSF) and maintained at room temperature for at least an additional 30 min before start of the experiment. At the start of the experiment slices were transferred to a submersion chamber (Warner Instruments, Hamden, CT) on a Siskiyou 4080P fixed-stage system (Grants Pass, OR) with a continuous flow rate of 2–3 mL/min aCSF saturated with 95% O_2_/5% CO_2_ at 32 ± 1 °C using an inline heater (Warner SC-20, Hamden, CT). Slices were visualized using an Axio Examiner A1 microscope (Zeiss, Thornwood, NY) equipped with a 40x water-immersion objective coupled with an infrared differential interference contrast and an integrated Dodt gradient camera system. Recordings were taken from mPFC pyramidal neurons of layer 2/3, including the PL and IL regions, and were identified as being approximately 50–250 μm from the slice midline and possessing a pyramidal neuron morphology. Patch recording pipettes with ~5 MΩ resistance were fabricated using a PC-10 puller (Narishige, Greencale, NY). For the whole-cell patch-clamp recordings the intracellular recording solution contained in mM unless otherwise stated: 115 K-gluconate, 10 HEPES, 5 KCl, 2 MgCl_2_, 2 Mg-ATP, 2 Na_2_-ATP, 0.4 Na_2_GTP and 10 Na_2_-phosphocreatine (pH 7.33). Recordings from mPFC neurons were amplified and filtered at 2 kHz (MultiClamp 700B amplifier, Axon Instruments, Union City, CA), and digitized with a sampling rate of 10 kHz (Digidata 1550A, Axon Instruments). Spontaneous currents were recorded at resting membrane potential (~75 ± 5 mV). Therefore, inward currents are considered excitatory amino acid currents (EPSCs), whereas the outward currents (inhibitory postsynaptic currents, IPSCs) were blocked by bath application of bicuculline (10 μM). Drugs, such as PF3845 (1 μM) were administered by bath application and synaptic currents were collected for 5 min for each experimental condition. Access resistance (<25 MΩ) was regularly monitored during recordings, and cells were rejected if resistance changed >15% during the experiment. Off-line analysis of synaptic currents was performed using the Minianalysis software (Version 6.0.8; Synaptosoft, Decatur, GA). Data for PF3845 conditions are presented as PF3845-induced change (**Δ**) on sEPSC frequency and sEPSC amplitude, indicating percent inhibition from control (control is set to 0%). Quantitative analyses were performed on 14–26 neurons per group.

### Statistical Analysis

All descriptive statistics are reported as means (*M*) ± standard error of the mean (*SEM*). All statistical analyses were conducted using one-way or two-way analysis of variances (ANOVAs) with Sex (2 levels: male, female) and/or Genotype [2 levels: Tat(−), Tat(+)] as factors, followed by Tukey’s post hoc tests if necessary. An alpha level of *p* < .05 was considered significant for all statistical tests used. Additionally, for the PF3845-induced inhibition data on sEPSCs a one-sample *t*-test was conducted to determine whether PF3845 significantly inhibited currents from percent control. When analyzing each group separately multiple comparisons were controlled for by dividing the alpha level by the number of comparisons. The relationship between inhibitory control and CB_1_R expression was assessed by Pearson correlation and simple linear regression analyses. Simple linear regression analysis was conducted to determine if observed changes in CB_1_R expression was significantly predictive of the behavioral measure inhibitory control. Effect sizes are reported using *ω*^2^ to best represent population effect size using an unbiased estimate (Yiğit and Mendes 2018).

## Results

### Behavioral Testing

**Fig. 2:**
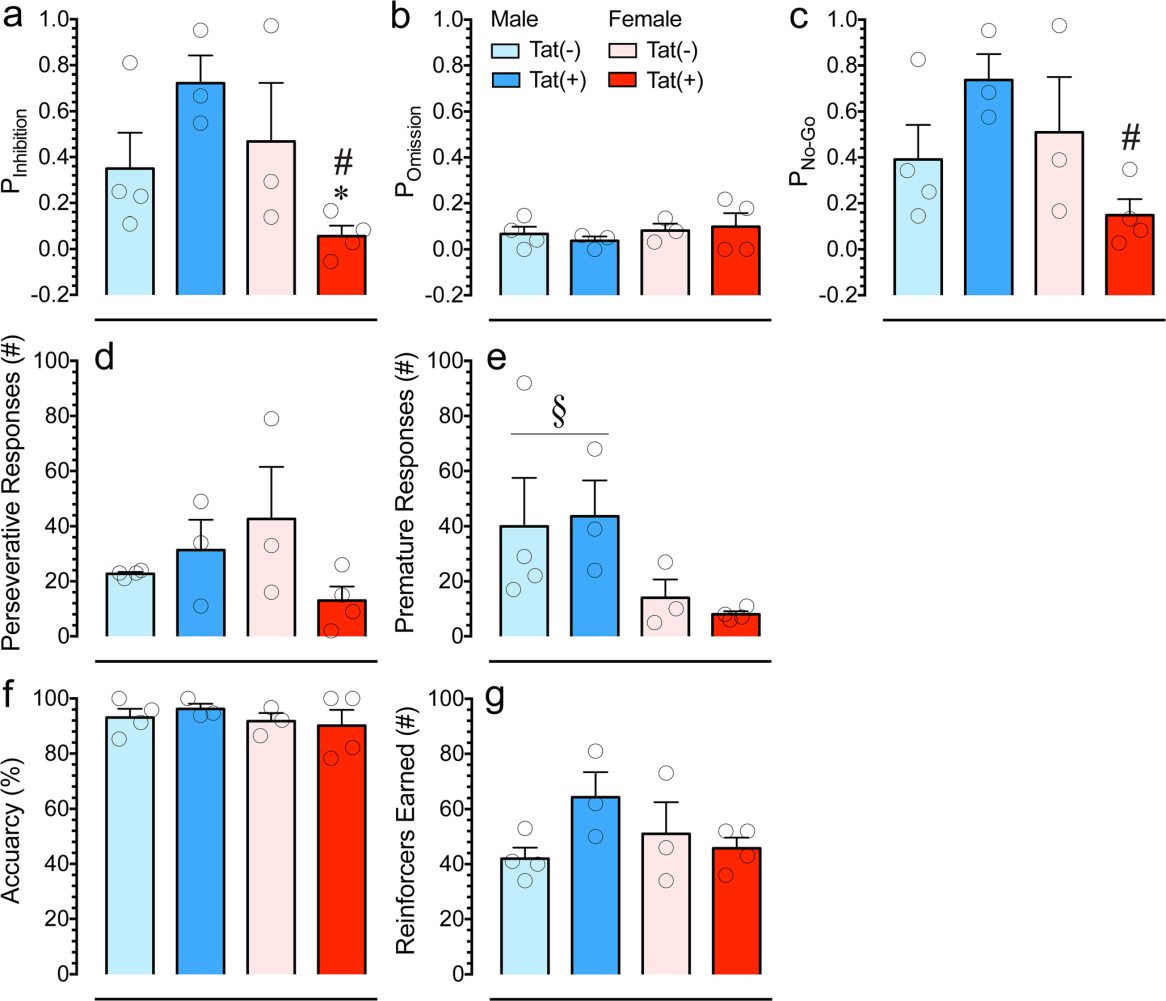
Details results from the Go/No-Go operant task. In all panels, data is organized, from left to right, as male Tat(−), male Tat(+), female Tat(−), and female Tat(+). **(a)** P_Inhibition_ indicates the probability that a subject will correctly inhibit its behavior when signaled (No-Go trials) while remaining sensitive to incorrect omissions (Go trials) and is obtained through the formula: P_Inhibition_ = (P_No-Go_-P_Omission_)/(1- P_Omission_). Using this calculation, P_Inhibition_ provides data on inhibitory control, or the ability of the subject only to omit responses when appropriate. When P_Inhibition_ = 1, the subject performed perfectly and only omitted responses during the No-Go trials; when P_Inhibition_ = 0, the subject either did not omit any responses or omitted every response. Data demonstrate a significant Sex by Genotype interaction on inhibitory control (^**#**^*p* = .028) with female Tat(+) mice showing poorer inhibitory control compared to male Tat(+) subjects [*p = .048 vs. male Tat(+)]. **(b)** P_Omission_ indicates the proportion of incorrect omissions during go trials to the total number of go trials. Values closer to 0 indicate lower incorrect omissions than values closer to 1. Data demonstrate that subjects in all groups show a low likelihood that they will incorrectly inhibit a nosepoke on a go trial. No significant differences were noted between groups. **(c)** P_No-Go_ indicates the proportion of correct omissions during no-go trials to the total number of no-go trials. Values indicate the likelihood that a subject will correctly omit a response on a no-go trial with values closer to 0 indicating a lesser likelihood than values closer to 1. A significant Sex by Genotype interaction was noted on P_No-Go_ (^**#**^*p* = .038) with female Tat(+) mice showing less correct omissions on No-Go trials compared to male Tat(+) subjects. **(d)** Perseverative responses across both trial types. Perseverative responses are nosepokes into the Start stimulus after the response requirement for beginning the trial has already been met. No significant differences were noted between groups. **(e)** Premature responses across both trial types. Premature responses are responses made into the Go stimulus before the Go stimulus is presented. A significant main effect of Sex (^§^*p* = .028) was noted on premature responding with males making more premature responses than females. **(f)** Mean accuracy responses across all Go trials for the test session without any significant differences between groups. **(g)** Mean reinforcers earned across all Go trials for the test session without any significant differences between groups

Results from behavioral testing are summarized in Fig. 2. A two-way ANOVA revealed a significant interaction between Sex and Genotype on P_Inhibition_, *F* (1, 10) = 6.61, *p* = .028, *ω*^2^ = .27 (Fig. 2a). Specifically female Tat(+) mice (*M* = .06, *SEM* = .05) demonstrated poorer inhibitory control than male Tat(+) mice (*M* = .72, *SEM* = .12; *p* = .048). There was no significant main effect of Sex or Genotype on P_Inhibition_ (all *p*’s > .05). When considering the results for P_Inhibition_, it is important to remember that this score derives from inhibition on both Go and No-Go trials and that a low score can stem from suppression of operantly trained behavior on Go trials, as recorded by P_Ommision_, or from a lack of inhibition on No-Go trials, as recorded by P_No-Go_. When considering scores for P_Ommision_ no significant main effect of Sex or Genotype was noted, nor was there a significant interaction between Sex and Genotype (Fig. 2b; all *p*’s > .05). Importantly, there was a significant interaction of Sex and Genotype on P_No-Go_, *F* (1, 10) = 5.70, *p* = .038, *ω*^2^ = .24 (Fig. 2c), with Tat expression inducing less inhibitory responses on No-Go trials in females in contrast to male mice. There was no significant main effect of Sex or Genotype on P_No-Go_ (all *p*’s > .05). When considered together, this pattern of results indicates that the differences seen in P_Inhibition_ scores are due to a failure of low scorers to inhibit on No-Go trials and not because of a performance deficit on Go trials. Thus we can interpret the low scorers as exhibiting poorer inhibitory control compared to high scorers.

Additionally, a two-way ANOVA examining differences in premature responses based on Sex and Genotype revealed a significant main effect of Sex on premature responses, *F* (1, 10) = 6.57, *p* = .028, *ω*^2^ = .31 (Fig. 2e). Specifically male mice (*M* = 41.57, *SEM* = 10.58) performed more premature responses than female mice (*M* = 10.57, *SEM* = 2.85). There was no significant main effect of Genotype nor a significant interaction between Sex and Genotype of premature responses (all *p*’s > .05). Furthermore, on perseverative responses no significant main effect was noted for Sex or Genotype, nor a significant Sex by Genotype interaction (Fig. 2d; all *p*’s > .05).

Lastly, two variables served as diagnostics to identify if any subject failed to perform or learn the task, including reinforcers earned and accuracy. A two-way ANOVA indicated that all animals reliably performed the task with no significant effects found for Sex and/or Genotype on number of reinforcers earned (Fig. 2f; all *p*’s > .05) or accuracy (Fig. 2g; all *p*’s > .05). Notably each group earned >40 reinforcers and had >80% accuracy at test indicating no disruptions in trained operant behavior during test phase.

### Immunohistochemistry

**Fig. 3:**
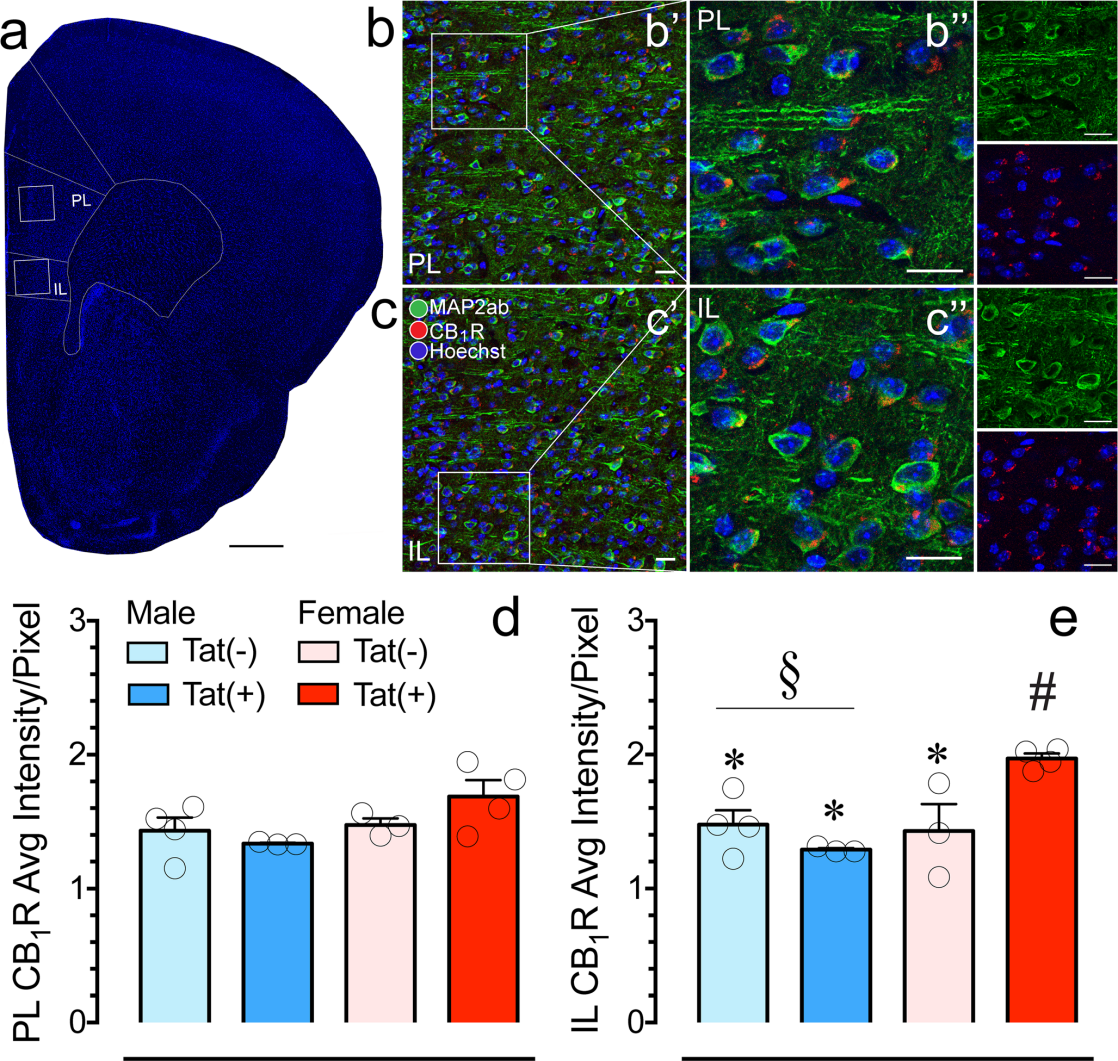
Shows the immunohistochemistry data on CB_1_R expression. **(a)** A representative brain slice (left hemisphere) of a Tat transgenic mouse stained for Hoechst (blue) with outlining the regions of interest, the PL and IL in the mPFC. **(b)** Higher magnification images of the PL region at 20x (**b’**) and 63x (**b”**) show staining for MAP2ab (green) and endogenous CB_1_R (red), with CB_1_Rs being specifically localized in the soma of mPFC neurons. **(c)** Similarly, higher magnification images of the IL region at 20x (**c’**) and 63x (**c”**) show staining for MAP2ab (green) and endogenous CB_1_R (red). **(d)** CB_1_R expression is quantified by averaging the intensity of the CB_1_R staining per pixel in the PL. No significant differences were noted between groups. **(e)** Quantification of CB_1_R expression in the IL. There was a significant main effect of Sex (^§^*p* = .015) and a Sex by Genotype interaction for CB_1_R intensity per pixel (^**#**^*p* = .007). Specifically, female Tat(+) animals had higher CB_1_R intensity compared to any of the other three groups [*p < .05 vs. female Tat(+)]. Scale bars: 20 μm. PL: prelimbic, IL: infralimbic, mPFC: medial prefrontal cortex

Results from immunohistochemistry staining examining differences in CB_1_R density for the PL and IL regions of the mPFC are summarized in Fig. 3. A two-way ANOVA on the PL region revealed no significant main effect of Sex or Genotype, nor a significant interaction between Sex and Genotype (Fig. 3d; all *p*’s > .05). In contrast, a two-way ANOVA on the IL region revealed a significant main effect for Sex, *F* (1, 10) = 8.55, *p* = .015, *ω*^2^ = .21, as well as a significant Sex by Genotype interaction, *F* (1, 10) = 11.36, *p* = .007, *ω*^2^ = .29. Specifically, female Tat(+) mice (*M* = 1.97, *SEM* = .04) showed greater CB_1_R expression compared to any other group (Fig. 3e; female Tat(−) mice: *M* = 1.43, *SEM* = .20; *p* = .023; male Tat(−) mice: *M* = 1.48, *SEM* = .11; *p* = .026; male Tat(+) mice: *M* = 1.29, *SEM* = .01; *p* = .006).

### Relationship between Inhibitory Control and CB_1_R Expression

**Fig. 4:**
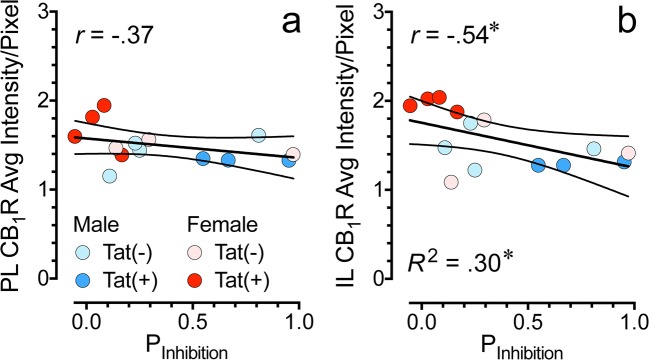
Shows the relationship between inhibitory control assessed in the Go/No-Go task (P_Inhibition_) and CB_1_R expression assessed by immunohistochemistry in the mPFC. **(a)** A Pearson correlation indicates no significant correlation between P_Inhibition_ and CB_1_R expression in the PL region of the mPFC. **(b)** For the IL a significant negative correlation was noted between P_Inhibition_ and CB_1_R expression (*r = −.543, *p* = .045), suggesting observed deficits in inhibitory control are associated with an upregulation in CB_1_R expression in the IL. A simple linear regression model demonstrates that CB_1_R expression is able to explain 30% of the total variance of inhibitory control assessed in the Go/No-Go task (*R^*2*^ = .295, *p* = .045). PL: prelimbic, IL: infralimbic, mPFC: medial prefrontal cortex

Results of the relationship between PFC-related behavioral inhibitory control and CB_1_R expression in the PL and IL regions are summarized in Fig. 4. A Pearson correlation demonstrated no significant correlation between P_Inhibition_ and CB_1_R expression in the PL region (*r* = −.371, *p* = .191; Fig. 4a) but a significant negative correlation for the IL region (*r* = −.543, *p* = .045; Fig. 4b), indicating an association between inhibitory control deficits and an upregulation of CB_1_R expression in the IL of the mPFC. The significant correlation between inhibitory control and CB_1_R expression in the IL region was further assessed by a simple linear regression analysis using IL CB_1_R expression as a predictor variable. Results indicate predictability of P_Inhibition_ by CB_1_R expression in the IL region accounting for 30% of total variance of inhibitory control [*R*^*2*^ = .295; *F*(1, 12) = 5.01, *p* = .045].

### Western Blot Analysis

**Fig. 5:**
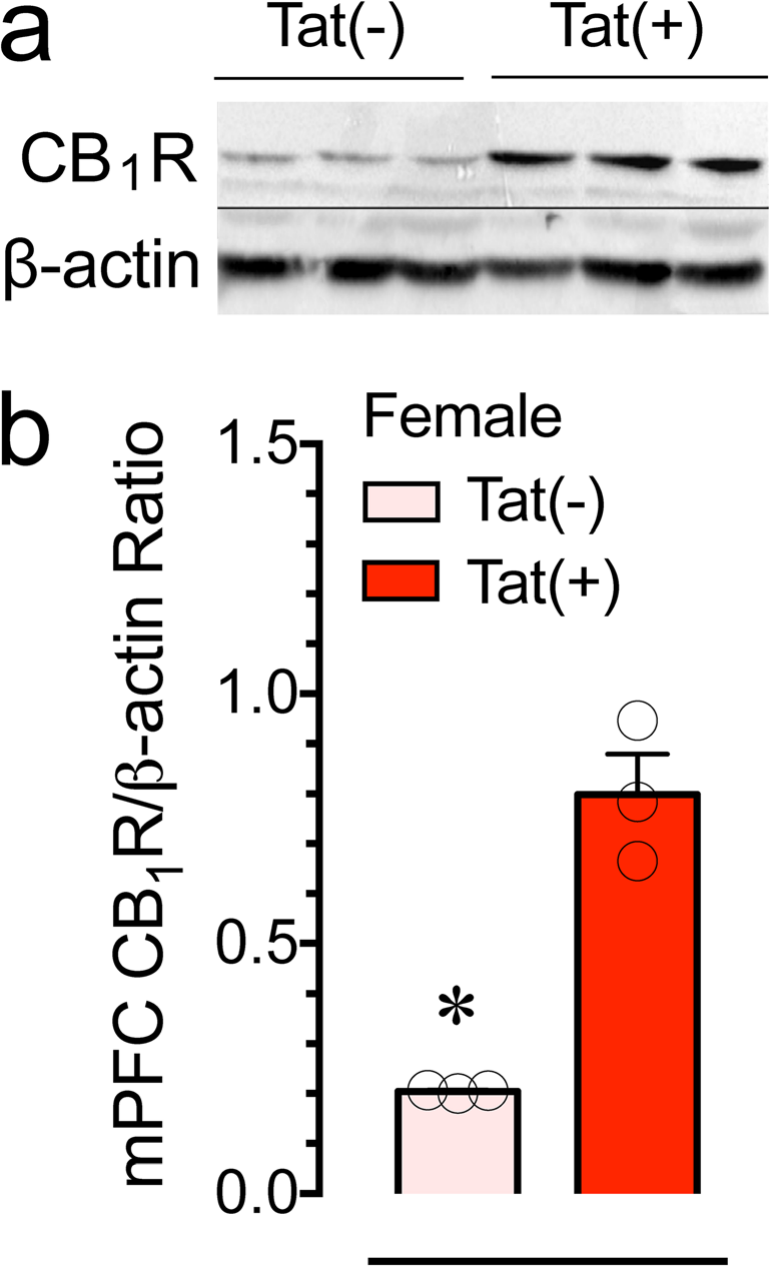
Shows the western blot data on CB_1_R protein expression in the mPFC of female Tat transgenic mice. **(a)** Shows representative immunoblots for CB_1_R and β-actin. **(b)** Quantification of CB_1_R protein expression indicated a significant main effect of Genotype (^*^p = .002) with female Tat(+) animals demonstrating increased CB_1_R protein levels compared to female Tat(−) controls. mPFC: medial prefrontal cortex

Results for the female Tat transgenic mice from western blot analysis for CB_1_R protein expression are summarized in Fig. 5. A one-way ANOVA revealed a significant main effect of Genotype on CB_1_R protein expression, *F* (1, 4) = 53.17, *p* = .002, *ω*^2^ = .90 (Fig. 5b). Specifically, female Tat(+) mice (*M* = .80, *SEM* = .08) demonstrated a significant upregulation of CB_1_R protein expression compared the female Tat(−) control mice (*M* = .20, *SEM* = .00).

### Electrophysiology

**Fig. 6:**
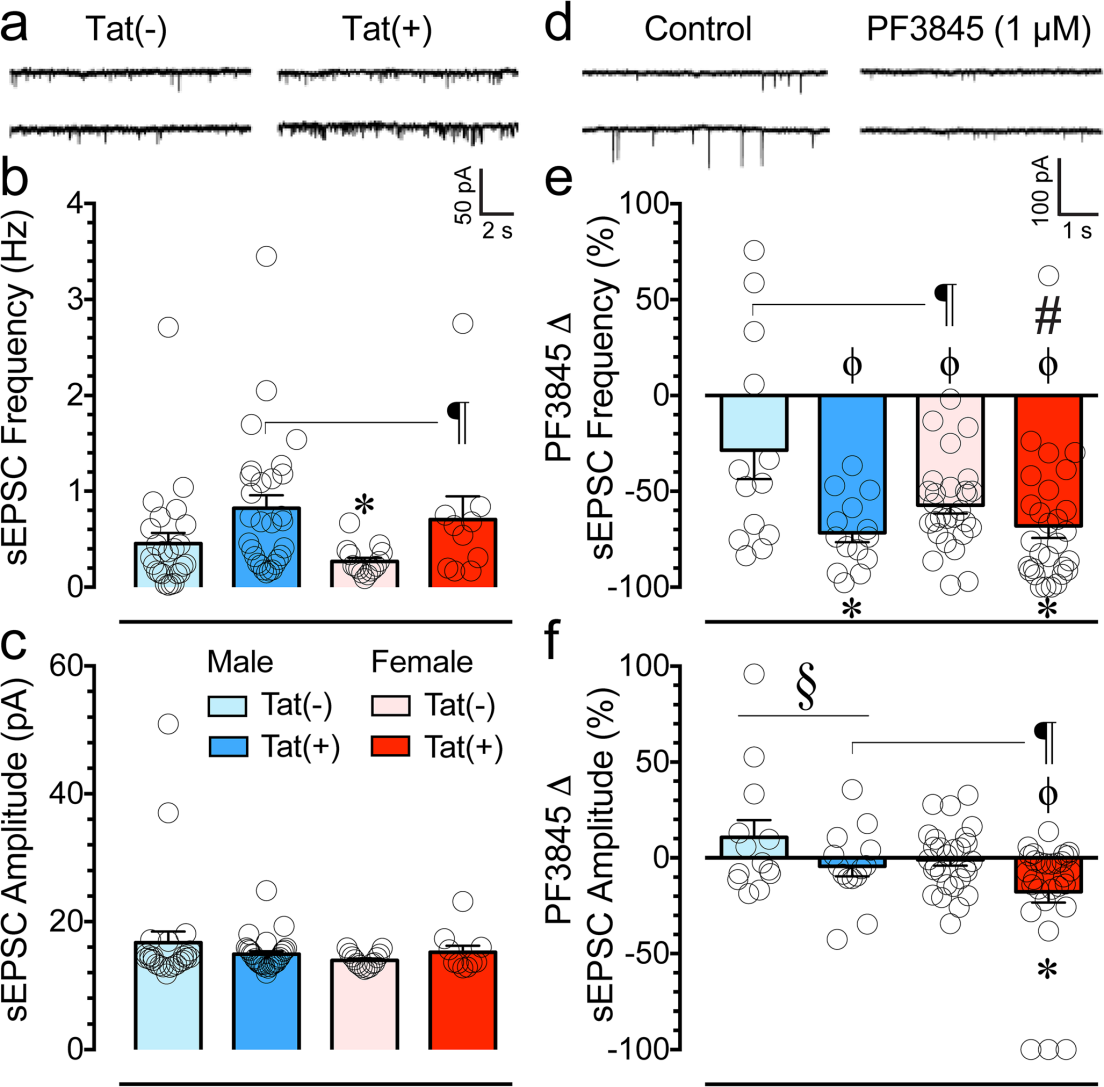
Details the electrophysiology data on spontaneous excitatory postsynaptic currents (sEPSCs) assessed in the mPFC in the presence and absence of PF3845 (1 μM) application. **(a)** Representative traces show sEPSCs on PFC brain slices for a control Tat(−) mouse and a transgenic Tat(+) subject. **(b)** A significant main effect of Genotype (^¶^*p* = .008) was noted on sEPSC frequency [*p = .026 vs. male Tat(+)]. **(c)** No significant differences on sEPSC amplitude were noted between groups. **(d)** Representative traces show sEPSCs on mPFC brain slices of a male transgenic Tat(+) subject before and after PF3845 (1 μM) bath application. **(e)** A significant effect was noted when applying PF3845 (1 μM) to the bath with PF3845 significantly inhibiting percent sEPSC frequency for all groups except for male Tat(−) mice [^ϕ^*p* < .001 vs. control response (0%) for each of the corresponding groups]. Additionally a significant main effect of Genotype (^¶^*p* = .001) was noted on sEPSC frequency as well as a significant Sex by Genotype interaction (^**#**^*p* = .039) with male Tat(−) mice showing less inhibition by PF3845 compared to male Tat(+) mice and female Tat(+) subjects [*p < .05 vs. male Tat(+)]. **(f)** For the PF3845-induced change on sEPSC amplitude a significant effect was noted, with PF3845 significantly inhibiting percent sEPSC amplitude only for female Tat(+) mice [^ϕ^*p* = .016 vs. control response (0%) of female Tat(+) subjects]. Further a significant main effect of Sex (^§^*p* = .038) was noted on PF3845-induced percent inhibition as well as a significant main effect of Genotype [^¶^*p* = .009; *p = .006 vs. male Tat(−)]. mPFC: medial prefrontal cortex

Results from electrophysiology recordings are summarized in Fig. 6. A two-way ANOVA revealed a significant main effect of Genotype on sEPSC frequency, *F* (1, 75) = 7.38, *p* = .008, *ω*^2^ = .07 (Fig. 6b). Specifically, Tat(+) mice (*M* = .79, *SEM* = .12) showed increased sEPSC frequency compared to Tat(−) mice (*M* = .38, *SEM* = .07). Additionally, a significant difference was noted between female Tat(−) mice (*M* = .27, *SEM* = .04) and male Tat(+) mice (*M* = .82, *SEM* = .13; *p* = .026). There was no significant main effect of Sex or Sex by Genotype interaction on sEPSC frequency (all *p*’s > .05). No significant main effects or interaction were noted on sEPSC amplitude (all *p*’s > .05; Fig. 6c).

For bath application of PF3845 (1 μM) a one-sample *t*-test demonstrated a significant inhibitory effect of PF3845 on percent sEPSC frequency, *t*(83) = −15.27, *p* < .001 (Fig. 6e). Separate one-sample *t*-tests demonstrated PF3845-induced percent inhibition for all groups except for male Tat(−) mice [male Tat(+): *t*(13) = −14.55, *p* < .001; female Tat(−): *t*(27) = −13.33, *p* < .001; female Tat(+): *t*(28) = −10.72, *p* < .001]. A two-way ANOVA revealed a significant main effect of Genotype on percent inhibition of sEPSC frequency by PF3845, *F* (1, 80) = 12.21, *p* = .001, *ω*^2^ = .12. Specifically, Tat(+) mice (*M* = −69.24, *SEM* = 4.54) showed more inhibition by PF3845 compared to Tat(−) mice (*M* = −48.18, *SEM* = 5.91). Additionally, there was a significant interaction between Sex and Genotype on percent inhibition of sEPSC frequency, *F* (1, 80) = 4.40, *p* = .039, *ω*^2^ = .04. Specifically male Tat(−) mice (*M* = −28.53, *SEM* = 15.01) demonstrated less inhibition by PF3845 compared to male Tat(+) mice (*M* = −71.67, *SEM* = 5.01; *p* = .006) and compared to female Tat(+) mice (*M* = −68.07, *SEM* = 6.35; *p* = .003).

For PF3845-induced change on percent sEPSC amplitude, a one-sample *t*-tests revealed significant PF3845-induced inhibition for female Tat(+) mice only, *t*(28) = −3.11, *p* = .016 (Fig. 6f). A two-way ANOVA revealed a significant main effect of Sex, *F* (1, 80) = 4.45, *p* = .038, *ω*^2^ = .04. Specifically, female mice (*M* = −9.43, *SEM* = 3.41) showed more inhibition by PF3845 than male mice (*M* = 2.87, *SEM* = 5.24). Additionally, there was a significant main effect of Genotype on percent inhibition of sEPSC amplitude, *F* (1, 80) = 7.22, *p* = .009, *ω*^2^ = .07. Specifically, Tat(+) mice (*M* = −13.31, *SEM* = 4.25) showed more inhibition by PF3845 compared to Tat(−) mice (*M* = 2.73, *SEM* = 3.58). There was no significant Sex by Genotype interaction on percent inhibition of sEPSC amplitude (*p* > .05). Additionally, a significant difference was noted between male Tat(−) mice (*M* = 10.67, *SEM* = 9.07) and female Tat(+) mice (*M* = −17.63, *SEM* = 5.67; *p* = .006).

## Discussion

The purpose of the current study was to investigate the behavioral profiles of Tat transgenic mice in a PFC-related operant conditioning task. Specifically in the post-cART era, a sharp increase has been observed in cortical cognitive deficiencies (Hardy and Vance 2009; Scott et al. 2011) with HIV-1 patients displaying more problems in executive function, memory consolidation, and inhibitory control, thus involving the PFC (Cysique et al. 2004; Garvey et al. 2009; Heaton et al. 2011). Our main objective was to demonstrate that Tat expression would lead to observable inhibitory control deficits as a result of prolonged Tat exposure. While it does not appear that Tat expression alone can account for differences in inhibitory control, the vulnerability towards prolonged Tat exposure seems to be sex-dependent. Results from the GNG task demonstrated increased inhibitory control deficits in female Tat(+) mice on P_Inhibition_ compared to their male counterparts. This holds true despite results on premature responding that did not mirror the pattern of inhibitory control deficits demonstrated by P_Inhibition_ data, but indicated a sex effect with males emitting more premature responses compared to females. In past literature, premature responding has been referred to as a measure of impulsivity, which is best captured through use of timeouts in the experimental design to implement a negative consequence to incorrect responses (Bari et al. 2008; Finn et al. 1999; Robbins 2002). As in the current study the use of timeouts slowed acquisition of the task to a near standstill, we removed timeouts for the reported GNG task. Therefore, premature responses in our study may be better characterized as an index of hyperactivity rather than impulsivity. Hyperactivity has been shown to be more prominent in males in a disease state compared to females (Acharjee et al. 2014; van den Buuse et al. 2017). Nevertheless, future studies should attempt to fade in timeouts for various incorrect responses potentially after the task has been acquired to help Tat transgenic mice learn the task.

The inhibitory control deficits demonstrated by P_Inhibition_ in our female Tat(+) mice are somewhat surprising as various past studies have reported higher vulnerability of male transgenic Tat(+) mice in different tasks, including rotarod activity and foregrip strength (Hahn et al. 2015b), and anxiety-like behaviors (Paris et al. 2014b). The lower vulnerability of females has suggested to be related to the documented neuroprotective role of classic steroid hormones estrogen (Hoffman et al. 2006; Paris et al. 2016; Tang et al. 1996) and its induced expression of anti-oxidants (Kumar et al. 2011; Rao et al. 2011). Additionally, human literature also supports the notion of male sex being a risk factor for increasing severity of HAND symptoms (Joska et al. 2011; Liu et al. 1996). Nevertheless it should be noted that studies focusing on HAND in populations exposed to resource-limited environments reported women to exhibit higher rates and intensity of symptoms compared to men (Chiesi et al. 1996; Gupta et al. 2007; Hestad et al. 2012; Wojna et al. 2006). It is suggested that an interaction between environmental stress and immune function might moderate the relationship between sex and severity of symptoms. In the current study, food restriction of our mice could potentially have served as a source of stress (Jensen et al. 2013; Yadawa and Chaturvedi 2016). Future studies may want to look at indicators of stress in the brain in an attempt to correlate levels of stress with severity of inhibitory control deficits.

The HIV-1 rodent literature is not devoid of studies that have shown female rodents to exhibit greater deficits in other domains of behavior on operant conditioning tasks. Most of these studies point to sex differences, with females showing a higher intensity of inflammatory responses in the hippocampus and therefore neuroinflammation serves as a key factor in accounting for these differences (Devi et al. 2010; Schwarz and Bilbo 2012; Zhang et al. 2008). There is a need for further research to investigate sex differences in PFC neuroinflammation, but available research points to greater expression of proinflammatory cytokines in female mice following intermediate ethanol exposure (Pascual et al. 2017). Sex differences in neuroinflammation are the likely cause of sex differences in signal detection task performance in HIV-1 transgenic rats; a recent study demonstrated that female HIV-1 transgenic rats were slower to acquire a signal detection task and had lower accuracy at test compared to males (McLaurin et al. 2017). Furthermore, while overall response rates were much lower for female HIV-1 transgenic rats, female mice specifically had lower correct rejections, which is akin to the lower P_No-Go_ scores characterized in the current study. While the exact neural mechanisms contributing to differences in performance on this task require additional exploration, neuroinflammation is a mechanism that has been demonstrated to potentially account for observed behavioral deficits in a number of disorders including HAND (Appay and Sauce 2008; Boska et al. 2014; Sas et al. 2007). Furthermore, the contribution of Tat to neuroinflammation in the CNS to promote neuronal injury and synaptic dysfunction has been reported in multiple studies in Tat transgenic mouse models (Hahn et al. 2015b; Paris et al. 2015; Paris et al. 2016) and other preclinical in vivo rodent models, e.g. Tat injections (Marker et al. 2013; Puccini et al. 2015). Even though an enhanced state of glial activation and additional evidence of impaired neurogenesis in the striatum appears to be specifically upregulated in male Tat(+) mice compared to females (Hahn et al. 2015a; Hahn et al. 2015b) further studies need to investigate the sex-dependent effects for neuroinflammation in the PFC.

As the eCB system has attracted interest as a target for treatment of neurodegenerative disorders, such as Parkinson’s disease and Alzheimer’s disease (Pertwee 2014; Scotter et al. 2010), we were interested in investigating the role of the eCB system, which regulates both immune function and cognition, as a potential target for treatment of HAND. When focusing on CB_1_R expression our immunohistochemistry data indicated that female Tat(+) mice who exhibited the poorest ability of inhibitory control in turn had the greatest expression of CB_1_R levels in the IL mPFC. We were able to confirm this significant upregulation of CB_1_R expression at the protein level by western blot analysis with female Tat(+) mice indicating significant higher CB_1_R protein expression levels in the mPFC compared to their control Tat(−) counterparts. An upregulation of CB_1_Rs has been reported in other experimental disease models, such as Parkinson’s disease (Brotchie 2003) as well as in brain tissue of HIV-1 infected patients with encephalitis (Cosenza-Nashat et al. 2011). Our data showed a correlation between the degree of IL CB_1_R upregulation and deficits on the GNG task, specifically on the ability of our subjects to perform an alternative to the Go response on No-Go trials, indicating IL machinery was disrupted in those animals with the highest levels of CB_1_R expression. The IL mPFC is important for modifying previously learned associations allowing the organism to emit alternative behaviors once task parameters become ambiguous (Mukherjee and Caroni 2018). In our study, inhibition on No-Go trials represents an alternative behavior to the Go response as the same Go stimulus is present on both trial types. In this way, disrupted functionality of this region accounts for the decreased probability a subject would emit the alternative response on a No-Go trial. Further experiments are needed to determine if the upregulation of CB_1_Rs observed in female Tat(+) mice contributes to the deficits seen in the GNG task or whether this upregulation is a compensatory response to the observed Tat-induced inhibitory control deficits in this group. Upregulation of cannabinoid receptors is seen in a number of other neurodegenerative disorders, such as Parkinson’s disease and Alzheimer’s disease, however their role is disease progression is still unclear (Benito et al. 2003; Farkas et al. 2012; Lastres-Becker et al. 2001; Lee et al. 2010; Ramirez et al. 2005; Van Laere et al. 2009; Westlake et al. 1994). Nevertheless, even though our data support the notion of an upregulation in CB_1_R expression, decreased or no change in CB_1_R expression has also been reported in a disease state depending on the brain region involved (Bedse et al. 2014; Benito et al. 2005; Silverdale et al. 2001). Further, as brain inflammation is a common feature in HIV-1 infection, an upregulation of CB_2_R appears to play an important role in HIV-1 encephalitis and simian immunodeficiency virus (SIV) (Benito et al. 2005; Cosenza-Nashat et al. 2011). While CB_1_R is abundantly expressed in the cortex specifically on pyramidal neurons, CB_2_R is expressed sparingly throughout the cortex but the greatest population exists on glial cells (Benito et al. 2008; Marsicano and Lutz 1999; Tsou et al. 1998). Quantitative image analysis of brains from HIV-1 infected patients with encephalitis indicated significant increases in CB_2_R expression in microglia, astrocytes and perivascular macrophages (Cosenza-Nashat et al. 2011). Despite the conflicting literature on changes in CB_1_R and CB_2_R expression levels in disease, it is agreed that the eCB system is a key modulator of synaptic function and is implicated in the progression of neurodegenerative disorders.

For synaptic function, the current study was able to demonstrate a significant disruption of synaptic function in the mPFC as indicated by the increased sEPSC frequency in Tat(+) mice compared to their Tat(−) counterparts (males and females). Enhanced glutamatergic neurotransmission by Tat has been demonstrated in previous in vitro studies (Brailoiu et al. 2008; Green and Thayer 2016; Musante et al. 2010; Xu et al. 2017) and is suggested to be mediated via Tat’s actions on NMDARs (Eugenin et al. 2007; Green and Thayer 2016; Raybuck et al. 2017) as well as non-NMDARs (Nath et al. 1996), leading to calcium influx, oxidative stress, and neuronal injury (Kaul et al. 2001). The Tat-induced increases in excitability, with the enhancement of the sEPSCs frequency but not affecting their amplitude has been reported recently in vitro (Xu et al. 2017) and suggests a presynaptic stimulatory effect on glutamate release (Brailoiu et al. 2008). Interestingly, CB_1_R stimulation has been demonstrated to limit glutamate-mediated synaptic excitation (Rossi et al. 2011). The involvement of the CB_1_Rs located on glutamatergic terminals is critical for the neuroprotective effects of eCBs in counteracting excitotoxicity (Chiarlone et al. 2014). It is known that upon excessive glutamate release, eCBs are produced perisynaptically to engage with presynaptic CB_1_Rs to inhibit excitatory transmission thus buffering against the excitotoxic effects of NMDAR activity in the postsynaptic neuron (Chiarlone et al. 2014; Huang et al. 2001). Various studies have shown that CB_1_Rs mediate the neuroprotective effects that eCBs exhibit against excitotoxic damage and inflammation (Shen and Thayer 1998; Xu et al. 2017). However, even though the activation of CB_1_Rs is known to be neuroprotective, therapeutic use of direct CB_1_R agonist is limited due to the pervasive psychoactive side effects associated with CB_1_R agonists that include sensorimotor, affective and cognitive disturbances (Di Marzo 2008). Additionally, given the speed at which eCBs are broken down, administration of eCB ligands is sure to have only a transient impact as they are immediately degraded by their corresponding eCB-metabolizing enzymes (Blankman and Cravatt 2013). Thus, neuroprotective effects can be achieved using eCB enzyme inhibitors, such as inhibiting the main AEA-metabolizing enzyme fatty acid amide hydrolase (FAAH), as demonstrated in a recent in vitro primary PFC neuron culture study in the context of Tat-toxicity (Hermes et al. 2018). Indeed, in the present study by using the FAAH enzyme inhibitor PF3845, which acts on CB_1_R-related mechanisms, Tat-induced increase on sEPSCs frequency was attenuated when bath applied, with Tat(+) brain slices being affected significantly more compared to Tat(−) brain slices (male and females). These results point towards neuroprotective effects by modulating eCB enzyme activity, potentially due to upregulation of AEA levels by PF3845. Noteworthy, in the context of Tat toxicity the upregulation of eCB signaling by PF3845 has been shown to not only dependent on CB_1_R-dependent mechanisms but also CB_2_R involvement (Hermes et al. 2018). Additionally, FAAH enzyme inhibition can also elevate non-cannabinoid lipid mediators (e.g., palmitoylethanolamide (PEA) and oleoylethanolamide (OEA)), which produce anti-inflammatory effects through a non-CR-mediated mechanism of action (i.e., peroxisome proliferator-activated receptor alpha). Despite the finding that PF3845 involves more than just CB_1_R-related mechanisms, our current study indicates the possibility for future studies to modulate the eCB system in vivo and examine the influence on Tat-induced behavioral deficits.

### Conclusion

In conclusion, the results of the present study indicate that the GNG task is a viable method to assess inhibitory control deficits associated with HAND in the Tat transgenic mouse model. Female Tat(+) animals showed greater inhibitory control deficits which is most likely due to sex differences in inflammatory responses to excitotoxic injury. These deficits are associated with an upregulation of CB_1_R. Further experiments are required to determine whether the observed CB_1_R upregulation is a compensatory response to Tat induced excitotoxicity or is contributing to the deficit as a part of disease pathogenesis. Tat also leads to an increase in sEPSCs, which may indicate a mechanism for how Tat eventually results in excitotoxic injury. These increases in sEPSC are attenuated by an eCB enzyme inhibitor, PF3845. Taken together, these results indicate a viable strategy to address damage precipitated by Tat may come in the form of therapies that modulate activity of the eCB system through use of enzyme inhibitors.

## Electronic supplementary material

ESM 1 (DOCX 28.7 kb)
