# Supplementary material for: Inhibitory Control Deficits Associated with Upregulation of CB1R in the HIV-1 Tat Transgenic Mouse Model of Hand
Source: J Neuroimmune Pharmacol. 2019 Aug 1;14(4):661–78. doi: 10.1007/s11481-019-09867-w (PMC6898753; doi:10.1007/s11481-019-09867-w)
Supplement: Supplementary file 1 — (DOCX 28.7 kb) [file 11481_2019_9867_MOESM1_ESM.docx]

Journal of Neuroimmune Pharmacology

INHIBITORY CONTROL DEFICITS ASSOCIATED WITH UPREGULATION OF CB_1_R IN THE HIV-1 TAT TRANSGENIC MOUSE MODEL OF HAND

**Supplemental Materials**

Ian R. Jacobs^a,^*, Changqing Xu^a^, Douglas J. Hermes^a^, Callie Xu^a^, Micah J. Niphakis^b^, Benjamin F. Cravatt^b^, Ken Mackie^c^, Aron H. Lichtman^d^, Bogna M. Ignatowska-Jankowska^e^ and Sylvia Fitting^a,^*

^a^Department of Psychology & Neuroscience, University of North Carolina at Chapel Hill, Chapel Hill, NC 27599, USA; ^b^Department of Chemical Physiology, Scripps Research Institute, La Jolla, CA 92037, USA; ^c^Department of Psychological & Brain Science, Indiana University, Bloomington, IN 47405, USA; ^d^Department of Pharmacology & Toxicology, Virginia Commonwealth University, Richmond, VA 23298, USA; ^e^Okinawa Institute of Science and Technology, Neuronal Rhythms in Movement Unit, Okinawa 904-0495, Japan

***Corresponding Authors:**

Ian R. Jacobs, M.A. and Sylvia Fitting; Ph.D., Phone: 919-962-6595; E-mail: [jacobsir@email.unc.edu](mailto:jacobsir@email.unc.edu) and [sfitting@email.unc.edu](mailto:sfitting@email.unc.edu)

**2. METHODS**

**2.3 Behavioral Training**

Training took place over four phases adapted from procedures described in previous literature (Gubner et al., 2010; Loos et al., 2010): phase 1: shaping the nose-poke response, phase 2: shaping the go response, phase 3: titrating the limited hold (LH), and phase 4; testing phase. Unless otherwise stated, advancement from a phase was contingent on criteria requiring the subject to earn 40 reinforcers on 2 consecutive days and an accuracy of 80%. Subjects advanced through the phases individually as they independently reach criteria. This advancement style ensures that all subjects receive the same relative training and prevents overtraining. Additionally, the intertrial interval (ITI) used in all phases was 10 s. The house light was illuminated 1 s before the start of the trial and is terminated once the animal retrieves a reinforcer. All sessions terminated after 30 min or 100 reinforcers were earned.

At ~6 weeks of age, mice were separated and individually housed to prevent fighting due to food deprivation. Over the next week, subjects were allowed to acclimate to their new living conditions and on the final two days, weights were taken and averaged to compute the initial weights from which the 85% target weight could be derived. At ~7 weeks of age, mice were gradually transitioned from ad libitum feeding to a restricted diet to lower their body weights to 85% of initial. At ~8 weeks of age mice entered the study and began phase 1.

Phase 1 started with magazine training in which a pellet was dispensed on a variable-time 2 min schedule to train the animal where reinforcement was delivered and familiarized them with the sound of a pellet being dispensed. If the subject consumes all pellets dispensed, then it moved on to the process of shaping the nosepoke response. The center port, which would later serve as the start stimulus, and the port immediately adjacent on the left or right side, which would serve as the go stimulus, on the nosepoke wall were illuminated. A single poke into either of these ports results in reinforcement. Pokes into other ports (during the ITI or after reinforcement has been delivered) did not result in reinforcement or punishment. Once subjects met criteria for this phase, they advanced to phase 2.

Phase 2 introduced the chain of behaviors necessary to receive reinforcement on a Go trial and also the No-Go trial on 20% of the trials. Go and No-Go trials were counterbalanced within group and the following description accounts for one of those two arrangements. A Go trial first required the subject to make an observing response into the start stimulus to begin a trial. Then, after a 2s trace period, the go stimulus illuminated and the subject was required to make a single poke into that port to receive reinforcement. On a No-Go trial, after completing the observing response, the house light flashed during the 2s trace, serving as the No-Go stimulus, before the go stimulus illuminates. Once the go stimulus is illuminated, the house light continues flashing for 1s before both terminate. If the animal made no pokes into any ports, they receive reinforcement. Just like in phase 1, other than not receiving reinforcement for not poking into a port during a No-Go trial, no action was taken if the subject emits an incorrect response. If the subject does poke during a No-Go trial, the trial and house light are terminated and the ITI is reset. Once subjects meet criteria for this phase, they advance to phase 3.

Phase 3 proceeded exactly as phase 2 with a few notable exceptions. The go reaction time (GoRT) was collected which was the latency from when the go stimulus illuminates to when the subject performs a nosepoke into that port. Once GoRTs were gathered, an individually titrated LH was introduced on the Go and No-Go stimuli. The LH is obtained through rank-ordering the GoRTs for an individual animal, then applying the 90^th^ percentile GoRT to the stimuli. In other words, the subject was required to respond to the Go stimulus, or withhold a response in the presence of the No-Go stimulus, in the LH period equal to the 90^th^ percentile of their GoRT in order to receive reinforcement. By adding the individually titrated LH, we were able to record omissions to the Go stimulus which would be important for calculating an index of inhibition. If a subject incorrectly omitted a Go response, the ITI was reset and the trial and house light were terminated. Subjects that met criteria for this phase advanced to phase 4, which is outlined in the behavioral training section (2.3.) in the manuscript.

**References**

Bodyak N, Slotnick B (1999) Performance of mice in an automated olfactometer: odor detection, discrimination and odor memory. Chem Senses 24:637-645.

Carlson KS, Whitney MS, Gadziola MA, Deneris ES, Wesson DW (2016) Preservation of Essential Odor-Guided Behaviors and Odor-Based Reversal Learning after Targeting Adult Brain Serotonin Synthesis. eNeuro 3.

Donders FC (1969) On the speed of mental processes. Acta Psychol (Amst) 30:412-431.

Ellis R, Langford D, Masliah E (2007) HIV and antiretroviral therapy in the brain: neuronal injury and repair. Nat Rev Neurosci 8:33-44.

Frederick DE, Brown A, Tacopina S, Mehta N, Vujovic M, Brim E, Amina T, Fixsen B, Kay LM (2017) Task-Dependent Behavioral Dynamics Make the Case for Temporal Integration in Multiple Strategies during Odor Processing. J Neurosci 37:4416-4426.

Gaspard JC, Bauer GB, Mann DA, Boerner K, Denum L, Frances C, Reep RL (2017) Detection of hydrodynamic stimuli by the postcranial body of Florida manatees (Trichechus manatus latirostris). Journal of Comparative Physiology A 203:111-120.

Gubner NR, Wilhelm CJ, Phillips TJ, Mitchell SH (2010) Strain Differences in Behavioral Inhibition in a Go/No-go Task Demonstrated Using 15 Inbred Mouse Strains. Alcohol Clin Exp Res 34:1353-1362.

Koek W, Gerak LR, France CP (2015) Effects of amphetamine, morphine, and CP 55, 940 on Go/No-Go task performance in rhesus monkeys. Behav Pharmacol 26:481-484.

Loos M, Staal J, Schoffelmeer ANM, Smit AB, Spijker S, Pattij T (2010) Inhibitory control and response latency differences between C57BL/6J and DBA/2J mice in a Go/No-Go and 5-choice serial reaction time task and strain-specific responsivity to amphetamine. Behavioural Brain Research 214:216-224.

Malvaut S, Gribaudo S, Hardy D, David LS, Daroles L, Labrecque S, Lebel-Cormier M-A, Chaker Z, Coté D, De Koninck P, Holzenberger M, Trembleau A, Caille I, Saghatelyan A (2017) CaMKIIα Expression Defines Two Functionally Distinct Populations of Granule Cells Involved in Different Types of Odor Behavior. Current Biology 27:3315-3329.e3316.

Montijn JS, Olcese U, Pennartz CMA (2016) Visual Stimulus Detection Correlates with the Consistency of Temporal Sequences within Stereotyped Events of V1 Neuronal Population Activity. Journal of Neuroscience 36:8624-8640.

Swalve N, Smethells JR, Carroll ME (2016) Progesterone attenuates impulsive action in a Go/No-Go task for sucrose pellets in female and male rats. Horm Behav 85:43-47.
